# Sudden fatal bleeding from a uretero-arterial fistula combined with pre-existing uretero-colic and uretero-vaginal fistulas 7 years after a cervical cancer surgery: a case report

**DOI:** 10.1186/s40792-019-0642-5

**Published:** 2019-05-23

**Authors:** Hiroki Yamazaki, Toru Nakamura, Yoshiro Otsuki, Mitsuteru Tsuchiya, Takashi Hamano, Hiroshi Adachi

**Affiliations:** 1 Department of Gynecology, Seirei Hamamatsu General Hospital, 2-12-12 Sumiyoshi, Hamamatsu City, Shizuoka Japan; 2 Department of General Thoracic Surgery, Seirei Hamamatsu General Hospital, 2-12-12 Sumiyoshi, Hamamatsu City, Shizuoka Japan; 3 Department of Pathology, Seirei Hamamatsu General Hospital, 2-12-12 Sumiyoshi, Hamamatsu City, Shizuoka Japan; 4 Department of Radiology, Seirei Hamamatsu General Hospital, 2-12-12 Sumiyoshi, Hamamatsu City, Shizuoka Japan

**Keywords:** Uretero-arterial fistula, Uretero-genital fistula, Uretero-enteric fistula

## Abstract

**Background:**

Uretero-arterial fistulas (UAFs) are a rare cause of hematuria and could be fatal often due to a diagnostic delay despite recent advances in the treatment modalities.

**Case presentation:**

A 52-year-old woman with a history of advanced cervical cancer developed a fever and was diagnosed with a left uretero-colic fistula. She also had a uretero-vaginal fistula and suffered from repeated urinary tract infections over 6 years. While waiting for an elective colostomy, she developed sudden perineal bleeding and died 14 h after the onset. The autopsy findings revealed that bleeding from a newly developed UAF spreads out to the extracorporeal space through the pre-existing fistulas.

**Conclusions:**

Bleeding from a UAF complicated by other uretero-genital and/or uretero-enteric fistulas could proceed rapidly resulting in a fatal outcome because of a lack of a tamponade effect. Early recognition of a UAF in high-risk patients is crucial for a prompt diagnosis, which might lead to a treatment success.

## Background

Uretero-arterial fistulas (UAFs) are a rare cause of hematuria but could be life-threatening despite the recent advances in the therapeutic modalities. This may be due to the diagnostic difficulty and a lack of recognition of the disease. We report a fatal case of a UAF combined with pre-existing uretero-enteral and genital fistulas that proceeded rapidly.

## Case presentation

**Fig. 1:**
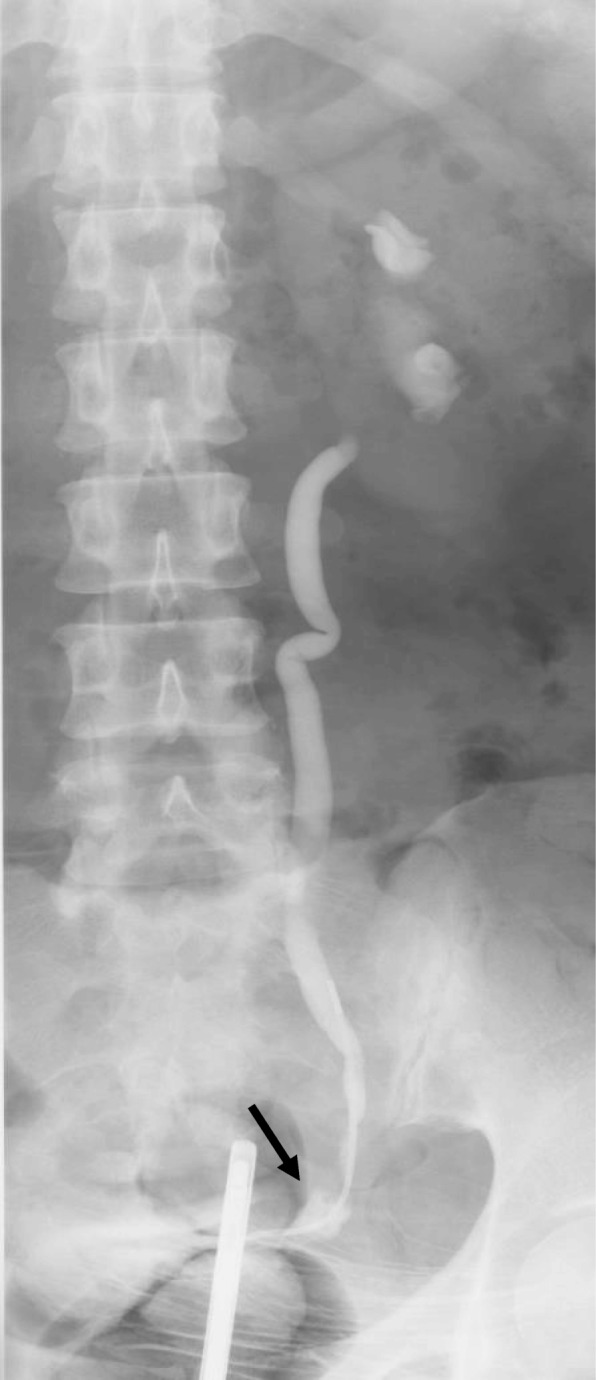
Retrograde pyelography revealed a left uretero-vaginal fistula (arrow)

**Fig. 2:**
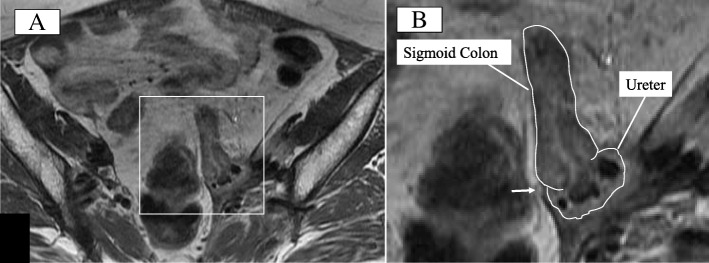
Magnetic resonance imaging revealed a fistula formation (arrow) between the sigmoid colon and ureter (**a** overall view and **b** enlarged view)

A 52-year-old woman was referred to the emergency room (ER) for a fever. She had undergone an extended hysterectomy for cervical cancer (stage IIb, pT2aN1M0) 7 years prior and developed a left uretero-vaginal fistula secondary to postoperative chemoradiotherapy managed with an indwelling ureteral stent (Fig. 1). Following that, she suffered from repeated urinary tract infections occasionally treated by antibiotics and required routine ureteral stent exchanges. She also had type 2 diabetes mellitus treated with pioglitazone hydrochloride over 6 years and revealed no signs of a cancer recurrence during that period. Magnetic resonance imaging revealed a fistula formation between her sigmoid colon and left ureter (Fig. 2). It was considered that a fecal leakage from the uretero-colic fistula worsened the urinary tract infection and an elective colostomy was planned.

One month after the diagnosis of the uretero-colic fistula while waiting for the colostomy, she visited the ER complaining of perineal bleeding. A physical examination revealed hypotension (systolic blood pressure 70 mmHg) and tenderness of her lower abdomen but without any bloody stool upon a rectal digital examination nor vaginal bleeding on colposcopy. The laboratory examination revealed an elevated C-reactive protein (CRP) level without anemia and microscopic hematuria and pyuria. She was diagnosed with urosepsis caused by a uretero-colic fistula and was admitted to the department of gynecology. Although she developed macroscopic hematuria after inserting a urinary catheter, the site of bleeding could not be identified by contrast-enhanced computed tomography (CT). Thirteen hours after the onset, her hypotension worsened resulting in hypovolemic shock (systolic blood pressure 40 mmHg) and massive genital bleeding became evident. She died 1 h later (14 h after the onset) despite intensive care.

**Fig. 3:**
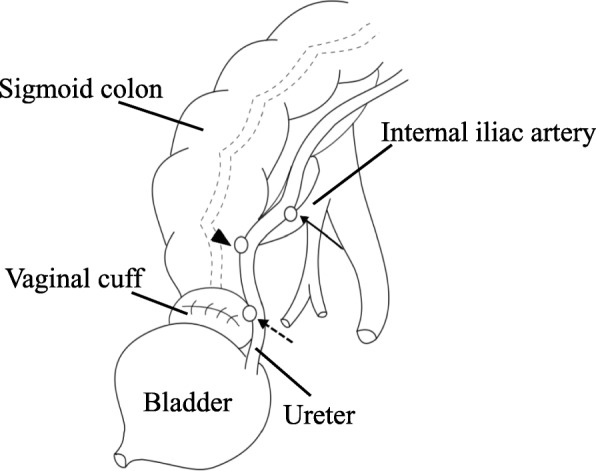
An autopsy revealed a newly developed fistula between the left ureter and iliac artery branch (arrow). Bleeding from this site might have spread out to the extracorporeal space through the pre-existing uretero-colic (arrowhead) and uretero-vaginal fistulas (dotted arrow)

**Fig. 4:**
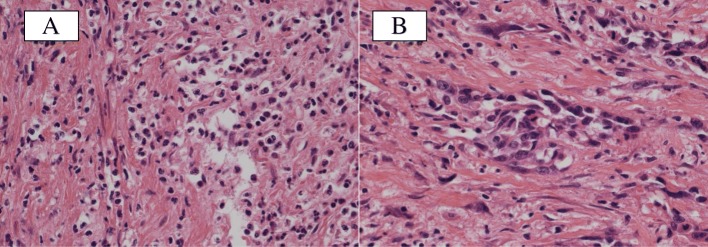
A histopathological examination revealed extensive inflammation and necrosis around the uretero-arterial fistula (**a** hematoxylin eosin staining) and an infiltrative squamous cell carcinoma in the surrounding tissue (**b** hematoxylin eosin staining)

The autopsy findings revealed a large amount of blood in the left renal pelvis, bladder, and sigmoid colon. In addition, the left internal iliac artery branch firmly adhered to the ureter and was found to form a UAF, which connected to the uretero-vaginal and uretero-sigmoid fistulas through the ureter (Fig. 3). These findings suggest that bleeding from the newly developed UAF spreads not only into the urinary tract but also over the genital and intestinal tracts through the pre-existing fistulas resulting in sudden death. A histopathological examination of the UAF revealed extensive inflammation with necrosis and an infiltrative squamous cell carcinoma (nonkeratinizing type) in the surrounding tissue (Fig. 4). Besides the previous surgery followed by chemoradiotherapy, her having suffered from repeated infections due to the indwelling ureteral catheter and the occult recurrent cancer tissue might have caused those histological changes that led to the fistula formation.

## Discussion

Since first being described in 1908 [1], over 100 reports on UAF have been published to date [2] and its incidence will increase due to more frequent use of indwelling ureteral catheters because of the recent increase in cancer survivors [3]. UAF can occasionally be fatal with a mortality rate of 20–30% even in the modern era [4–6]. Eighty-five percent of UAFs are reported to be a secondary etiology after pelvic surgery or radiation [7], and cervical cancer is one of the most frequent causes of the disease [2, 4]. Our case had several other risk factors for UAF, such as a history of a chronic indwelling ureteral stent, repeated urinary tract infections, and diabetes mellitus as comorbidities. Furthermore, the autopsy confirmed the local recurrence of cervical cancer, which had never been detected during the postoperative surveillance. Those combined risk factors might have caused chronic pressure between the ureter and left internal iliac artery branch resulting in histopathological ischemic changes. In addition, it was considered that the pre-existing uretero-colic and uretero-vaginal fistulas contributed to this sudden fatal outcome. Because the left ureter had already become connected to the extracorporeal space through these fistulas, bleeding from the newly developed UAF spreads out through that route without a tamponade effect. Therefore, a prompt diagnosis and treatment were quite essential especially in our case.

Endovascular management has been widely used as the main treatment strategy for UAFs over a traditional open repair [8–11] and also has been successfully applied to the treatment of arterio-enteric fistulas [12, 13]. It would be promising to enable immediate hemostasis with less morbidity than surgical treatment, especially in patients who had prior pelvic interventions and a vascular pathology.

Despite these recent advances in the treatment modalities, diagnosing UAF is still a clinical challenge, and a diagnostic delay has been reported to be associated with a significantly poor outcome [14–16].

Although contrast CT, cystoscopy, and standard angiography have been employed as diagnostic modalities, the clinical utility or sensitivity are far from satisfactory and the diagnosis is often made at the time of surgery [2, 15, 17]. In addition to the lack of awareness of the etiology, even the most sensitive angiography could often fail to visualize extravasation into the ureter due to intermittent bleeding and the presence of an indwelling ureteral stent [2, 18, 19]. To improve the diagnostic accuracy, pharmacological provocative maneuvers other than standard angiography have been provocative angiography in which the indwelling ureteral stent is removed to help visualize the fistula [6, 14, 18, 20]. Contrast-enhanced CT was not diagnostic even after the onset of the bleeding in our case and therefore angiography including provocative maneuvers might have helped obtain a correct diagnosis. However, because stent manipulation not only can be diagnostic but also cause massive bleeding [21, 22], provocative angiography should be attempted with simultaneous arterial embolization or surgical bypass grafting [6]. Further, it was considered that to suspect a UAF by itself was a key issue. Considering the history of pelvic interventions including chemoradiotherapy and chronic indwelling ureteral catheters requiring routine exchanges due to repeated urinary tract infections, we should have suspected the possibility of the UAF during the earlier stage of the workup in our case.

## Conclusions

Bleeding from UAFs complicated with other uretero-genital and/or uretero-enteric fistulas could proceed rapidly resulting in a fatal outcome. It was considered that both a diagnostic delay and concomitant fistula, which eliminated the tamponade effect against bleeding, were responsible for the sudden and fatal outcome in our case. Moreover, we should be aware of UAFs as the most critical cause of hematuria in high-risk patients and early recognition of the disease itself is a key issue both for the diagnostic and a successful treatment especially in cases with other ureteral fistulas.

## Abbreviations

CRP: C-reactive protein
CT: Computed tomography
ER: Emergency room
UAF: Uretero-arterial fistula

## References

[1] Moschcowitz AVIX (1908). Simultaneous ligation of both external iliac arteries for secondary hemorrhage. Ann Surg.

[2] van den Bergh RC, Moll FL, de Vries JP, Lock TM (2009). Arterioureteral fistulas: unusual suspects-systematic review of 139 cases. Urology..

[3] Fox JA, Krambeck A, McPhail EF, Lightner D (2011). Ureteroarterial fistula treatment with open surgery versus endovascular management: long-term outcomes. J Urol..

[4] Batter SJ, McGovern FJ, Cambria RP (1996). Ureteroarterial fistula: case report and review of the literature. Urology..

[5] McCullough MC, Oh EE, Lucci JA, Alvarez EA (2012). Ureteroarterial fistula. J Obstet Gynaecol..

[6] Vandersteen DR, Saxon RR, Fuchs E, Keller FS, Taylor LM, Barry JM (1997). Diagnosis and management of ureteroiliac artery fistula: value of provocative arteriography followed by common iliac artery embolization and extraanatomic arterial bypass grafting. J Urol.

[7] Bergqvist D, Parsson H, Sherif A (2001). Arterio-ureteral fistula--a systematic review. Eur J Vasc Endovasc Surg..

[8] Malgor RD, Oderich GS, Andrews JC, McKusick M, Kalra M, Misra S (2012). Evolution from open surgical to endovascular treatment of ureteral-iliac artery fistula. J Vasc Surg..

[9] Okada T, Yamaguchi M, Muradi A, Nomura Y, Uotani K, Idoguchi K (2013). Long-term results of endovascular stent graft placement of ureteroarterial fistula. Cardiovasc Intervent Radiol..

[10] Das A, Lewandoski P, Laganosky D, Walton J, Shenot P (2016). Ureteroarterial fistula: a review of the literature. Vascular..

[11] Subiela JD, Balla A, Bollo J, Dilme JF, Soto Carricas B, Targarona EM (2018). Endovascular management of ureteroarterial fistula: single institution experience and systematic literature review. Vasc Endovascular Surg..

[12] Baril DT, Carroccio A, Ellozy SH, Palchik E, Sachdev U, Jacobs TS (2006). Evolving strategies for the treatment of aortoenteric fistulas. J Vasc Surg..

[13] Leonhardt H, Mellander S, Snygg J, Lonn L (2008). Endovascular management of acute bleeding arterioenteric fistulas. Cardiovasc Intervent Radiol..

[14] Kerns DB, Darcy MD, Baumann DS, Allen BT (1996). Autologous vein-covered stent for the endovascular management of an iliac artery-ureteral fistula: case report and review of the literature. J Vasc Surg..

[15] Krambeck AE, DiMarco DS, Gettman MT, Segura JW (2005). Ureteroiliac artery fistula: diagnosis and treatment algorithm. Urology..

[16] Darcy M (2009). Uretro-arterial fistulas. Tech Vasc Interv Radiol..

[17] van den Bergh RC, Moll FL, de Vries JP, Yeung KK, Lock TM (2008). Arterio-ureteral fistula: 11 new cases of a wolf in sheep’s clothing. J Urol..

[18] Quillin SP, Darcy MD, Picus D (1994). Angiographic evaluation and therapy of ureteroarterial fistulas. AJR Am J Roentgenol..

[19] Coelho H, Freire MJ, Azinhais P, Temido P. Arterioureteral fistula: an unusual clinical case. BMJ Case Rep. 2016;2016. 10.1136/bcr-2016-214400 PMC4800223 26969358

[20] Dervanian P, Castaigne D, Travagli JP, Chapelier A, Tabet G, Parquin F (1992). Arterioureteral fistula after extended resection of pelvic tumors: report of three cases and review of the literature. Ann Vasc Surg..

[21] Cass AS, Odland M (1990). Ureteroarterial fistula: case report and review of literature. J Urol..

[22] Matsui Y, Fujikawa K, Oka H, Fukuzawa S, Takeuchi H (2001). Ureteroarterial fistula in a patient with a single functioning kidney. Int J Urol..

