# Correction to: Sudden fatal bleeding from a uretero-arterial fistula combined with pre-existing uretero-colic and uretero-vaginal fistulas 7 years after a cervical cancer surgery: a case report

**DOI:** 10.1186/s40792-019-0666-x

**Published:** 2019-07-04

**Authors:** Hiroki Yamazaki, Toru Nakamura, Yoshiro Otsuki, Mitsuteru Tsuchiya, Takashi Hamano, Hiroshi Adachi

**Affiliations:** 1 Department of Gynecology, Seirei Hamamatsu General Hospital, 2-12-12 Sumiyoshi, Hamamatsu City, Shizuoka Japan; 2 Department of General Thoracic Surgery, Seirei Hamamatsu General Hospital, 2-12-12 Sumiyoshi, Hamamatsu City, Shizuoka Japan; 3 Department of Pathology, Seirei Hamamatsu General Hospital, 2-12-12 Sumiyoshi, Hamamatsu City, Shizuoka Japan; 4 Department of Radiology, Seirei Hamamatsu General Hospital, 2-12-12 Sumiyoshi, Hamamatsu City, Shizuoka Japan

**Correction to: Surg Case Rep (2019) 5:85**

**https://doi.org/10.1186/s40792-019-0642-5**

In the original publication of this article [1], the family name of an author is wrong.

The correct name should be Mitsuteru Tsuchiya.

The original article has been corrected.
